# HIV epidemic in Russia and neighbouring countries

**DOI:** 10.7448/IAS.17.4.19502

**Published:** 2014-11-02

**Authors:** Vadim Pokrovskiy

**Affiliations:** Russian Federal AIDS Centre, Moscow, Russian Federation

## Abstract

Reports of HIV/AIDS cases attributed to sexual transmission from foreigners were published in the USSR in the mid of 80s. In the initial decade of the epidemic, the subtype B was found in men who have sex with men (MSM) population and several non-B subtypes were identified in heterosexual persons. The first case of HIV infection in intravenous drug users (IVDU) was reported in 1993 and since then a specific subtype A and its recombinants invaded the intravenous drug users (IVDU) populations of the region with the highest rate in Estonia, Russia and Ukraine. The concentrated HIV epidemic in IVDUs is still the main problem in the Eastern Europe; however, the rate of heterosexual transmission is increasing and many evidences of HIV prevalence rise in MSM are published. UNAIDS estimations for the number of HIV-positive persons living in the region range from 980,000 to 1,300,000 but distribution of HIV-cases is uneven and the prevalence rate of HIV infection in separate regions is over 1%. Mass seasonal labour migration from Central Asia and Caucasian republics to Russia transmits HIV to these countries. Prevention programs in the region are limited, and ART coverage is not more than 20% of the total HIV-positive population. The lack of concern about the epidemic, absence of effective national strategies and limited allocated resources are the main barriers to prevention and care in many countries. Local conflicts, rising religiosity and discrimination are adverse factors. The near-term forecast for the epidemic in the region is pessimistic and further international advocacy is needed to improve the situation.

